# Production of plasmid-encoding NDM-1 in clinical *Raoultella ornithinolytica* and *Leclercia adecarboxylata* from China

**DOI:** 10.3389/fmicb.2015.00458

**Published:** 2015-05-21

**Authors:** Fengjun Sun, Zhe Yin, Jiao Feng, Yefeng Qiu, Defu Zhang, Wenbo Luo, Huiying Yang, Wenhui Yang, Jie Wang, Weijun Chen, Peiyuan Xia, Dongsheng Zhou

**Affiliations:** ^1^Department of Pharmacy, Southwest Hospital, The Third Military Medical UniversityChongqing, China; ^2^State Key Laboratory of Pathogen and Biosecurity, Beijing Institute of Microbiology and EpidemiologyBeijing, China; ^3^Laboratory Animal Center, Academy of Military Medical SciencesBeijing, China; ^4^Beijing Institute of Genomics, Chinese Academy of SciencesBeijing, China

**Keywords:** *Raoultella ornithinolytica*, *Leclercia adecarboxylata*, NDM-1, plasmid, carbapenem resistance

## Abstract

*Raoultella ornithinolytica* YNKP001 and *Leclercia adecarboxylata* P10164, which harbor conjugative plasmids pYNKP001-NDM and pP10164-NDM, respectively, were isolated from two different Chinese patients, and their complete nucleotide sequences were determined. Production of NDM-1 enzyme by these plasmids accounts for the carbapenem resistance of these two strains. This is the first report of *bla*_NDM_ in *L. adecarboxylata* and third report of this gene in *R. ornithinolytica*. pYNKP001-NDM is very similar to the IncN2 NDM-1-encoding plasmids pTR3, pNDM-ECS01, and p271A, whereas pP10164-NDM is similar to the IncFII_Y_ *bla*_NDM-1_-carrying plasmid pKOX_NDM1. The *bla*_NDM-1_ genes of pYNKP001-NDM and pP10164-NDM are embedded in Tn*125*-like elements, which represent two distinct truncated versions of the NDM-1-encoding Tn*125* prototype observed in pNDM-BJ01. Flanking of these two Tn*125*-like elements by miniature inverted repeat element (MITE) or its remnant indicates that MITE facilitates transposition and mobilization of *bla*_NDM-1_ gene contexts.

## Introduction

*Raoultella ornithinolytica* is widely found in aquatic environments, insects and fishes. *R. ornithinolytica* is able to convert histidine to histamine (scombroid toxin) and is thus known to cause fish poisoning. Symptoms primarily manifest with facial flushing, dizziness, vomiting, diarrhea, dyspnea, headache, urticarial, and generalized pruritus and commonly subside in a few hours (Kanki et al., 2002). Infections by *R. ornithinolytica* are exceedingly rare in humans and have been reported as bloodstream, urinary tract and soft tissue infections in adults and as fatal neonatal infections. Most adult cases are linked with underlying diseases, especially malignancies (Morais et al., 2009; Mau and Ross, 2010; Solak et al., 2011; Hadano et al., 2012; Haruki et al., 2014; Chun et al., 2015).

*Raoultella ornithinolytica* produces at least two different chromosomally encoded class A β-lactamases. Accordingly, *R. ornithinolytica* is resistant to ampicillin but commonly remains susceptible to cefotaxime and imipenem (Walckenaer et al., 2004). Notably, carbapenem-resistant *R. ornithinolytica* has been reported due to the production of plasmid-encoding carbapenemase KPC-3 (Castanheira et al., 2009) or NDM-1 (Khajuria et al., 2013; Zhou et al., 2014).

*Leclercia adecarboxylata* is a ubiquitous organism that is rarely clinically isolated in humans. However, *L. adecarboxylata* has been recognized as an opportunistic pathogen in immunocompromised patients suffering from primary diseases and often depends on co-flora to cause polymicrobial infection (Shin et al., 2012; De Mauri et al., 2013; Garcia-Fulgueiras et al., 2014). *L. adecarboxylata* is isolated from various clinical specimens (e.g., blood, feces, sputum, urine, and wound pus) and causes bacteremia, endocarditis, sepsis, peritonitis, cellulitis, endocarditis, and cholecystitis (Shin et al., 2012; De Mauri et al., 2013; Garcia-Fulgueiras et al., 2014). *L. adecarboxylata*-induced monomicrobial infections (e.g., wound infection, pharyngeal abscess, and bacteremia) have also been reported in immunocompetent patients (Hess et al., 2008; Bali et al., 2013; Michael et al., 2013; Garcia-Fulgueiras et al., 2014; Keren et al., 2014).

*Leclercia adecarboxylata* is generally susceptible to commonly used antibiotics, but there are a few reports of *L. adecarboxylata* harboring different antibiotic resistance mechanisms. Cephalosporin- and carbapenem-resistant strains of *L. adecarboxylata* have been identified due to the production of extended-spectrum β-lactamase (ESBL) SHV-12 (Mazzariol et al., 2003) and carbapenemase KPC-2 (Geffen et al., 2013) or VIM-1 (Papagiannitsis et al., 2013), respectively. Notably, clinical isolates of multidrug-resistant *L. adecarboxylata* have been known to harbor multiple antibiotic resistance genes that are captured by class 1 integrons (Yao et al., 2011; Shin et al., 2012; Garcia-Fulgueiras et al., 2014).

NDM is an Ambler class B metallo-β-lactamase that confers resistance to nearly all β-lactam antibiotics, including carbapenems, and *bla*_NDM_ genes have been identified in a large array of *Enterobacteriaceae* species (Nordmann et al., 2011; Johnson and Woodford, 2013; Dortet et al., 2014). Both *R. ornithinolytica* and *L. adecarboxylata* are members of *Enterobacteriaceae*. In this study, we analyzed complete nucleotide sequences of two different NDM-1-encoding plasmids, pYNKP001-NDM, and pP10164-NDM, recovered from *R. ornithinolytica* YNKP001 and *L. adecarboxylata* P10164, respectively, of clinical origin in China.

## Materials and methods

### Bacterial strains and identification

The use of human specimens and all related experimental protocols were approved by the Committee on Human Research of indicated institutions and carried out in accordance with the approved guidelines. Informed consent was obtained from the indicated patients. All bacterial strains were subjected to species identification by BioMérieux VITEK 2, Bruker MALDI Biotyper, and 16S rRNA gene sequencing. For 16S rRNA gene sequence determination, nearly the complete coding region of 16S rRNA gene was amplified by PCR with the universal primers 27f (AGAGTTTGATCCTGGCTCAG) and 1492r (TACCTTGTTACGACTT) (Frank et al., 2008). The major carbapenemase and ESBL genes (Table S1) were subjected to PCR detection. All PCR amplicons were sequenced on an ABI 3730 Sequencer with the same primers for PCR.

### Plasmid conjugal transfer

Plasmid conjugal transfer experiments were carried out with *Escherichia coli* EC600 (rifampin-resistant) or TB1 (streptomycin-resistant) used as recipient and the *bla*_NDM_-positive strain YNKP001 or P10164 as donor. Then, 3 ml of overnight culture of each of donor and recipient bacteria were mixed together, harvested and resuspended in 80 μl of brain heart infusion (BHI) broth (BD Biosciences). The mixture was spotted on a 1-cm^2^ filter membrane placed on BHI agar (BD Biosciences) plates. The plates were incubated for mating at 37°C for 12–18 h. Bacteria were washed from filter membrane and spotted on Muller-Hinton (MH) agar (BD Biosciences) plates containing 1000 μg/ml rifampin or 250 μg/ml streptomycin and 200 μg/ml ampicillin for selection of *bla*_NDM_-positive *E. coli* transconjugants.

### Detection of carbapenemase activity

Activity of class A/B/D carbapenemases was determined via a CarbaNP test (Dortet et al., 2012) with modifications. Overnight bacterial cell culture in MH broth was diluted 1:100 into 3 ml of fresh MH broth, and bacteria were allowed to grow at 37°C with shaking at 200 rpm to reach an OD_600_ of 1.0–1.4. If required, ampicillin was used at 200 μg/ml. Bacterial cells were harvested from 2 ml of the above culture and washed twice with 20 mM Tris-HCl (pH 7.8). Cell pellets were resuspended in 500 μl of 20 mM Tris-HCl (pH 7.8) and lysed by sonication, followed by centrifugation at 10000 × g at 4°C for 5 min. Then, 50 μl of the supernatant (enzymatic bacterial suspension) was mixed with 50 μl of substrate I–V, followed by incubation at 37°C for a maximum of 2 h. Substrate I: 0.054% red phenol plus 0.1 mM ZnSO_4_ (pH 7.8). Substrate II: 0.054% red phenol plus 0.1 mM ZnSO_4_ (pH 7.8) and 0.6 mg/μl imipenem. Substrate III: 0.054% red phenol plus 0.1 mM ZnSO_4_ (pH 7.8), 0.6 mg/μl mg imipenem, and 0.8 mg/μl tazobactam. Substrate IV: 0.054% red phenol plus 0.1 mM ZnSO_4_ (pH 7.8), 0.6 mg/μl mg imipenem, and 3 mM EDTA (pH 7.8). Substrate V: 0.054% red phenol plus 0.1 mM ZnSO_4_ (pH 7.8), 0.6 mg/μl mg imipenem, 0.8 mg/μl tazobactam, and 3 mM EDTA (pH 7.8).

### Determination of minimum inhibitory concentration (MIC)

The MIC values of the indicated bacterial strains were tested by VITEK 2 according to the manufacturer's instructions, and antimicrobial susceptibility was assessed by the Clinical and Laboratory Standards Institute (CLSI) standards.

### Determination of plasmid DNA sequences

The chromosome DNA-free plasmid DNA was isolated from the cell cultures of indicated *E. coli* transconjugant using a Qiagen large construct kit and then sequenced using the whole-genome shotgun strategy in combination with Illumina HiSeq 2500 sequencing technology. The contigs were assembled with Velvet, and the gaps were filled through combinatorial PCR and Sanger sequencing with an ABI 3730 Sequencer. The genes were predicted with GeneMarkS and further annotated by BLASTP against UniPort and NR databases.

### Nucleotide sequence accession numbers

The complete sequences of plasmids pP10164-NDM and pYNKP001-NDM were submitted to GenBank under accession numbers KP900016 and KP900017, respectively.

## Results

### Characterization of *R. ornithinolytica* YNKP001 and *L. adecarboxylata* P10164

*Raoultella ornithinolytica* YNKP001 was recovered in November 2010 from the blood specimens of a 4-year-old child with acute encephalitis, bronchitis and tympanitis from a hospital in Kunming City in China. *L. adecarboxylata* P10164 was isolated in August 2012 from the sputum specimens of a 43-year-old male with pneumonia admitted to a teaching hospital in Chongqing City in China. Both patients received empirical intravenous administration with ceftazidime for at least 1 week, but their symptoms did not improve. Subsequent antimicrobial susceptibility tests indicated that both strains were resistant to multiple β-lactams, including imipenem, and meropenem, but remained susceptible to fluoroquinolones. The patients then received intravenous administration with moxifloxacin and were cured and discharged approximately 10 days after initiating antimicrobial treatment.

Plasmids pP10164-NDM and pYNKP001-NDM could be readily transferred from P10164 and YNKP001 into *E. coli* EC600 and TB1, respectively, generating two corresponding *E. coli* transconjugants: P10164-NDM-EC600 and YNKP001-NDM-TB1. PCR detection of the major ESBL and carbapenemase genes (Table S1) indicated that P10164 harbored the *bla*_NDM_, *bla*_CTX−M−1_ group, *bla*_TEM_, and *bla*_OXA−1_ group genes, whereas YNKP001, P10164-NDM-EC600 and YNKP001-NDM-TB1 contained only *bla*_NDM_, as confirmed by PCR amplicon sequencing. Class B carbapenemase activity was detected in P10164, YNKP001, P10164-NDM-EC600 and YNKP001-NDM-TB1 (Figure S1).

P10164 was resistant to all of the tested drugs, including penicillin, β-lactam inhibitor, cephalosporin, carbapenem, monobactam, fluoroquinolone, furane, and aminoglycoside (Table 1). YNKP001, P10164-NDM-EC600, and YNKP001-NDM-TB1 were resistant to penicillins, β-lactam inhibitors, cephalosporins, and carbapenems but remained susceptible to fluoroquinolones, aminoglycosides, aztreonam, and macrodantin (Table 1).

**Table 1:** **MIC values and antimicrobial susceptibility**.

**Category**	**Antibiotics**	**MIC (μg/ml)/antimicrobial susceptibility**					
		**P10164**	**P10164-NDM-EC600**	**EC600**	**YNKP001**	**YNKP001-NDM -TB1**	**TB1**
Penicillins	Ampicillin	≥32/R	≥32/R	16/I	≥32/R	≥32/R	16/I
	Ampicillin/sulbactam	≥32/R	≥32/R	8/S	≥32/R	≥32/R	8/S
	Piperacillin	≥128/R	≥128/R	≤4/S	≥128/R	≥128/R	≤4/S
	Piperacillin/tazobactam	≥128/R	≥128/R	≤4/S	≥128/R	≥128/R	≤4/S
Cephalosporins	Cefazolin	≥64/R	≥64/R	≤4/S	≥64/R	≥64/R	≤4/S
	Cefuroxime sodium	≥64/R	≥64/R	16/I	≥64/R	≥64/R	16/S
	Cefuroxime axetil	≥64/R	≥64/R	16/I	≥64/R	≥64/R	16/S
	Cefotetan	≥64/R	≥64/R	≤4/S	≥64/R	≥64/R	≤4/S
	Ceftriaxone	≥64/R	≥64/R	≤1/S	≥64/R	≥64/R	≤1/S
	Ceftazidime	≥64/R	≥64/R	≤1/S	≥64/R	≥64/R	≤1/S
Carbapenems	Imipenem	≥16/R	≥16/R	≤1/S	≥16/R	≥16/R	≤1/S
	Meropenem	8/R	4/R	≤0.25/S	≥16/R	≥16/R	≤0.25/S
Monobactams	Aztreonam	16/R	≤1/S	≤1/S	≤1/S	≤1/S	≤1/S
Fluoroquinolones	Ciprofloxacin	≥4/R	≤0.25/S	≤0.25/S	≤0.25/S	≤0.25/S	≤0.25/S
	Levofloxacin	≥8/R	0.5/S	0.5/S	0.5/S	≤0.25/S	≤0.25/S
Furanes	Macrodantin	≥512/R	≤16/S	≤16/S	32/S	≤16/S	≤16/S
Aminoglycosides	Amikacin	≥64/R	≤2/S	≤2/S	≤2/S	≤2/S	≤2/S
	Gentamicin	≥16/R	≤1/S	≤1/S	≤1/S	≤1/S	≤1/S
	Tobramycin	≥16/R	≤1/S	≤1/S	≤1/S	≤1/S	≤1/S

### Comparative genomics of PYNKP001-NDM

**Figure 1:**
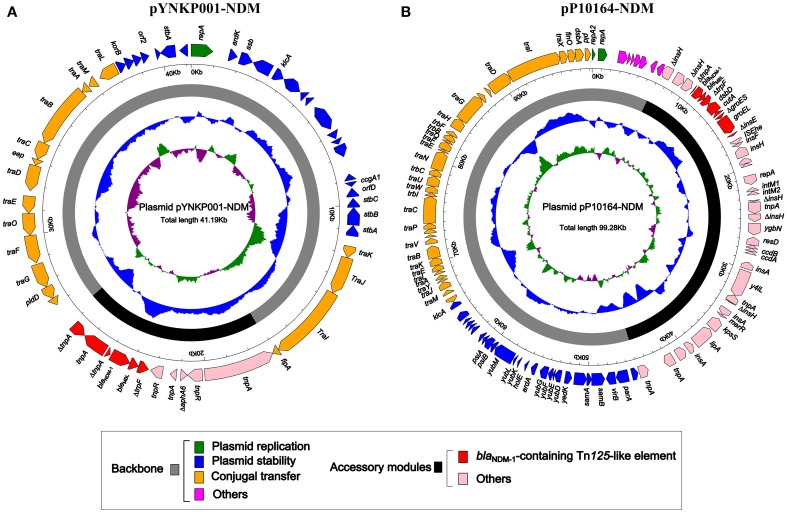
**Schematic maps of plasmids pYNKP001-NDM (A) and pP10164-NDM (B)**. Genes are denoted by arrows and colored based on gene function classification. The innermost circle presents GC-Skew [(G − C)/(G + C)] with a window size of 500 bp and step size of 20 bp. The blue circle presents GC content. Backbone and accessory module regions are also shown.

As revealed by high-throughput sequencing with a mean coverage fold of 143, plasmid pYNKP001-NDM was 41,190 bp in size with a mean GC content of 50.8% and contained 54 open reading frames (ORFs) (Figure 1A). The replication module present on pYNKP001-NDM belonged to the IncN2 incompatibility group (Poirel et al., 2011). This plasmid was mostly similar to another NDM-encoding IncN2 plasmid, pTR3 (Chen et al., 2012), with genetic differences in only three single nucleotide polymorphisms (SNPs).

**Figure 2:**
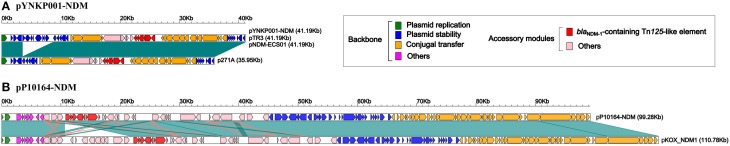
**Linear comparison of YNKP001-NDM (A) or pP10164-NDM (B) with its closely related plasmid**. Genes are denoted by arrows and colored based on gene function classification. Dark green shading denotes shared regions of homology (>98% nucleotide similarity).

Further linear genomic comparison (Figure 2A) was performed with all four NDM-encoding IncN2 plasmids, namely, pYNKP001-NDM, pTR3 (Chen et al., 2012), pNDM-ECS01 (Netikul et al., 2014), and p271A (Poirel et al., 2011). The former three plasmids essentially had the same genomic organization. The genome of each plasmid could be divided into the backbone and accessory module. The backbone was composed of genes responsible for plasmid replication (*repA*), stability (*stbABC*, *ssb*, *korB*, *klcA*, etc.) and conjugal transfer (*tra*). p271A lacked a 5.2 Kb region found in pYNKP001-NDM, pTR3, and pNDM-ECS01. This region corresponded to the CUP (conserved upstream repeat)-controlled regulon commonly found in the IncN1 and IncN2 plasmids, and the loss of this region might be due to recombination between CUPs (Partridge et al., 2012).

**Figure 3:**
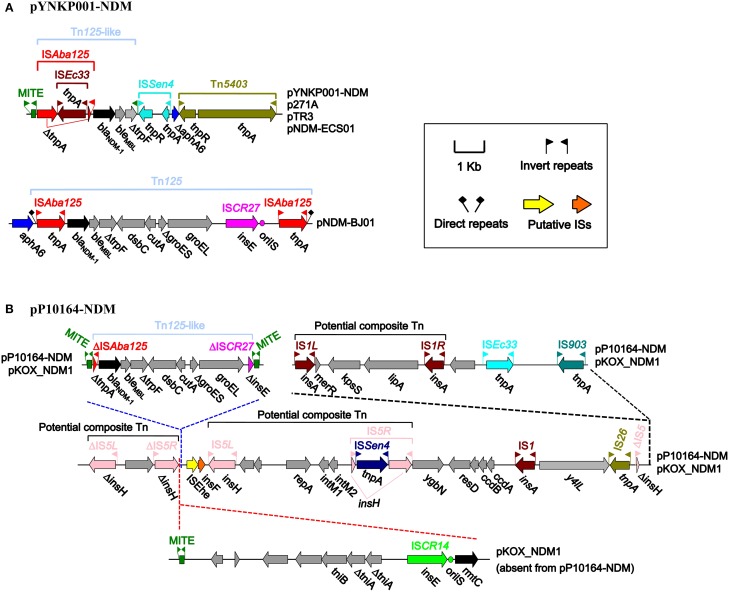
**Accessory modules of plasmids pYNKP001-NDM (A) and pP10164-NDM (B)**. Genes are denoted by arrows and colored based on gene function classification.

The four plasmids shared a single conserved accessory module, which was sequentially organized as an intact miniature inverted repeat element (MITE), a *bla*_NDM−1_-containing Tn*125*-like element, a MITE remnant, an intact IS*Sen4* element with 26 bp inverted repeats (IRs) at both sides, a truncated *aphA6* (aminoglycoside resistance) gene, and an intact Tn*5403* element with 39 bp IRs at both ends (Figure 3A). The Tn*125* prototype was sequentially organized as IS*Aba125*, *bla*_NDM−1_, *ble*_MBL_ (bleomycin resistance), *ΔtrpF*, *dsbC*, *cutA*, *ΔgroES*, *groEL*, *ISCR27*, and IS*Aba125*; Tn*125* was a typical composite transposon (Tn) containing two flanking copies of IS*Aba125* (each with 26 bp IRs at both ends); Tn*125* could be inserted into a site downstream of *aphA6*, leaving 3 bp direct repeats (DRs) at both ends, as observed in pNDM-BJ01 (Hu et al., 2012; Poirel et al., 2012) (Figure 3A). Compared with the Tn*125* prototype, the Tn*125*-like element in pYNKP001-NDM lacked the entire fragment of *dsbC*, *cutA*, *ΔgroES*, *groEL*, *ISCR27*, and IS*Aba125*. In addition, the upstream copy of IS*Aba125* was a truncated version lacking the left IR and was interrupted by IS*Ec33* (with 17 bp IRs at both ends) (Figure 3A).

The flanking of a genetic context (e.g., class 1 integron) by two identical MITEs has been recently characterized as a mechanism for mobilizing antimicrobial resistance determinants via MITE-mediated transposition or homologous recombination (Poirel et al., 2009; Domingues et al., 2013; Zong, 2014). Notably, the Tn*125*-like element in pYNKP001-NDM was embedded between an intact 257 bp MITE and a 55 bp MITE remnant (Figure 3A). The intact MITE contained 39 bp IRs and could potentially form a long stem-loop RNA structure (Delihas, 2011), whereas the downstream MITE remnant corresponded to the 3′-terminal 55 bp fragment (including the right IR) of the intact MITE.

### Comparative genomics of PP10164-NDM

Plasmid pP10164-NDM was fully sequenced, with a mean coverage fold of 248. Moreover, it was 99,276 bp in size, with a mean GC content of 55%, and contained 101 ORFs (Figure 1B). pP10164-NDM was assigned to the IncFII_Y_ incompatibility group encoding two different replication proteins IncFII_Y_ (*repA*) and IncFIB (*repB*) (Villa et al., 2010). Comparative genomics analysis was performed with the only two characterized NDM-encoding IncFII_Y_ plasmids: pP10164-NDM and pKOX_NDM1 (Huang et al., 2013) (Figure 2B). pP10164-NDM differed from pKOX_NDM1 in 214 SNPs, 11 single-nucleotide indels, and three large deletions (11285 bp, 180 bp, and 45 bp, respectively). pP10164-NDM and pKOX_NDM1 had very similar backbones composed of genes responsible for plasmid replication (*repA* and *repB*), stability (*pmaAB*, *psiAB*, *klcA*, *yub*, etc.) and conjugal transfer (*tra*); the 45 bp deletion (nucleotide position 102082 to 102126 in pKOX_NDM1; located within the *traD* gene) was the only observed structural difference between the backbones of pP10164-NDM and pKOX_NDM1 (Figure 2B).

pP10164-NDM contained a single accessory module that was 38,098 bp in size, in which the 11285 bp and 180 bp deletions (nucleotide positions 10620–21904 and 37157–37336 in pKOX_NDM, respectively) compared with the counterpart of pKOX_NDM1 were located (Figure 3B). The accessory module of pKOX_NDM1 harbored three copies of 256 bp MITEs highly similar to the above-mentioned 257 bp MITE, constituting a linear structure organized as the 11285 bp region (MITE plus 11029 bp region), MITE, Tn*125*-like element, and MITE (Figure 3B). Homologous recombination mediated by the first two copies of MITE appeared to lead to the insertion of the 11285 bp region into pKOX_NDM1 relative to pP10164-NDM (Figure 3B). In addition to the first copy of MITE, the 11285 bp region still contained *rmtC* (16S rRNA methylase for aminoglycoside resistance), IS*CR14* and an uncharacterized Tn (*tniB*, Δ*tniA*, and *tniA*) (Figure 3B).

The *bla*_NDM−1_-harboring Tn*125*-like element was flanked by the second and third copies of MITE, indicating a similar mechanism of MITE-mediated insertion to the Tn*125*-like element (Figure 3B). Compared with the Tn*125* prototype in pNDM-BJ01, the Tn*125*-like element in pP10164-NDM (Figure 3B) exhibited absence of a downstream copy of IS*Aba125*, as well as truncation of *ISCR27* and an upstream copy of IS*Aba125*.

In addition, three potential composite Tns, with the characteristic of being flanked by two separate copies of IS*5* or IS*1*, were identified within the accessory module of pP10164-NDM or pKOX_NDM1, but none of the flanking DRs as target sites could be found.

## Discussion

Horizontal transfer of plasmid-borne *bla*_NDM_ genes enabled NDM enzymes to be rapidly spread in *Enterobacteriaceae*, less frequently in *Acinetobacter*, and rarely in *Pseudomonas* (Nordmann et al., 2011; Johnson and Woodford, 2013; Dortet et al., 2014). *Klebsiella pneumoniae* and *E. coli* are the most commonly described *bla*_NDM_-carrying *Enterobacteriaceae* species, and *bla*_NDM_ genes have been described in many other enterobacterial species, such as *K. oxytoca*, *K. ozaenae*, *Citrobacter freundii*, *Enterobacter aerogenes*, *Enterobacter cloacae*, *Proteus mirabilis*, *Morganella morganii*, *Providencia spp*., *Serratia marcescens*, and *Salmonella enterica* (Ou et al., 2014; Biedenbach et al., 2015). These NDM-producing bacteria have been shown to cause hospital- and community-acquired infections (Ou et al., 2014; Biedenbach et al., 2015). *bla*_NDM−1_-positive bacteria are frequently found in environmental settings, indicating environmental origins of *bla*_NDM_ genes in human pathogens, whereas use of antimicrobial drugs in an indiscriminate manner in some countries makes *bla*_NDM−1_-positive bacteria spread easily and pose a serious public health threat (Walsh et al., 2011; Isozumi et al., 2012).

This is the first report of *bla*_NDM_ in *L. adecarboxylata*. The clinical *L. adecarboxylata* isolate characterized herein harbors a conjugative IncFII_Y_ plasmid pP10164-NDM that encodes the NDM-1 enzyme. pP10164-NDM is highly similar to pKOX_NDM1, which is recovered from a nosocomial *K. oxytoca* strain from a patient following medical transfer from a hospital in Jiangxi to Taiwan, China (Huang et al., 2013). There are only two reports of *bla*_NDM_ in *R. ornithinolytica*, one from India (Khajuria et al., 2013) and the other from China (Zhou et al., 2014), both of which were confined to surveillance of *bla*_NDM_ genes. This work presents extended evidence that the production of NDM-1 by a conjugative IncN2 plasmid pYNKP001-NDM accounts for the carbapenem resistance of a clinical *R. ornithinolytica* isolate from China. pYNKP001-NDM is very similar to pTR3 (identified in *K. pneumoniae* from a Chinese patient) (Chen et al., 2012), pNDM-ECS01 (in *E. coli* from a Thai patient) (Netikul et al., 2014) and p271A (in *E. coli* from a patient following medical transfer from a hospital in Bangladesh to Australia) (Poirel et al., 2011).

The *bla*_NDM−1_ genes of pYNKP001-NDM and pP10164-NDM are embedded in Tn*125*-like elements, which are characterized as two distinct truncated versions of the prototype NDM-encoding Tn*125*, as observed in pNDM-BJ01 (Hu et al., 2012).

The Tn*125*-like elements with a large array of derivatives represent the major genetic platforms for *bla*_NDM_ genes across host bacteria (Bonnin et al., 2012; Mcgann et al., 2012; Partridge and Iredell, 2012; Zhang et al., 2013; Zong and Zhang, 2013; Fiett et al., 2014; Jones et al., 2014; Mataseje et al., 2014). Notably, flanking of Tn*125*-like elements by MITE or its remnant as observed herein indicates that MITE facilitates transposition and mobilization of *bla*_NDM−1_ gene contexts.

### Conflict of interest statement

The authors declare that the research was conducted in the absence of any commercial or financial relationships that could be construed as a potential conflict of interest.

## Supplementary material

Figure S1 **Detection of carbapenemase activity**. In the presence of any carbapenemase, relevant carbapenems are hydrolyzed and transformed into its carboxylic form, leading to a pH decrease, which is detected by a color change of phenol red solution (red to yellow-orange). Ambler class A carbapenemases are at least partially inhibited by tazobactam, whereas class B carbapenemases (metallo-ß-lactamases) are inhibited by divalent cation chelators, such as EDTA. There is no available chemical inhibitor for class D carbapenemases. In this study, the *bla*_NDM−1_-positive strains *R. ornithinolytica* YNKP001 and *L. adecarboxylata* P10164, *E. coli* YNKP001-NDM-TB1 and P10164-NDM-EC600, and *K. pneumoniae* ATCC BAA-2146 (positive control) (Rasheed et al., 2013) exhibited class B carbapenemase activity. As expected, *E. coli* EC600 and DH10B had no carbapenemase activity.
