# Supplementary figures and images for: Production of plasmid-encoding NDM-1 in clinical Raoultella ornithinolytica and Leclercia adecarboxylata from China
Source: Front Microbiol. 2015 May 21;6:458. doi: 10.3389/fmicb.2015.00458 (PMC4439573; doi:10.3389/fmicb.2015.00458)

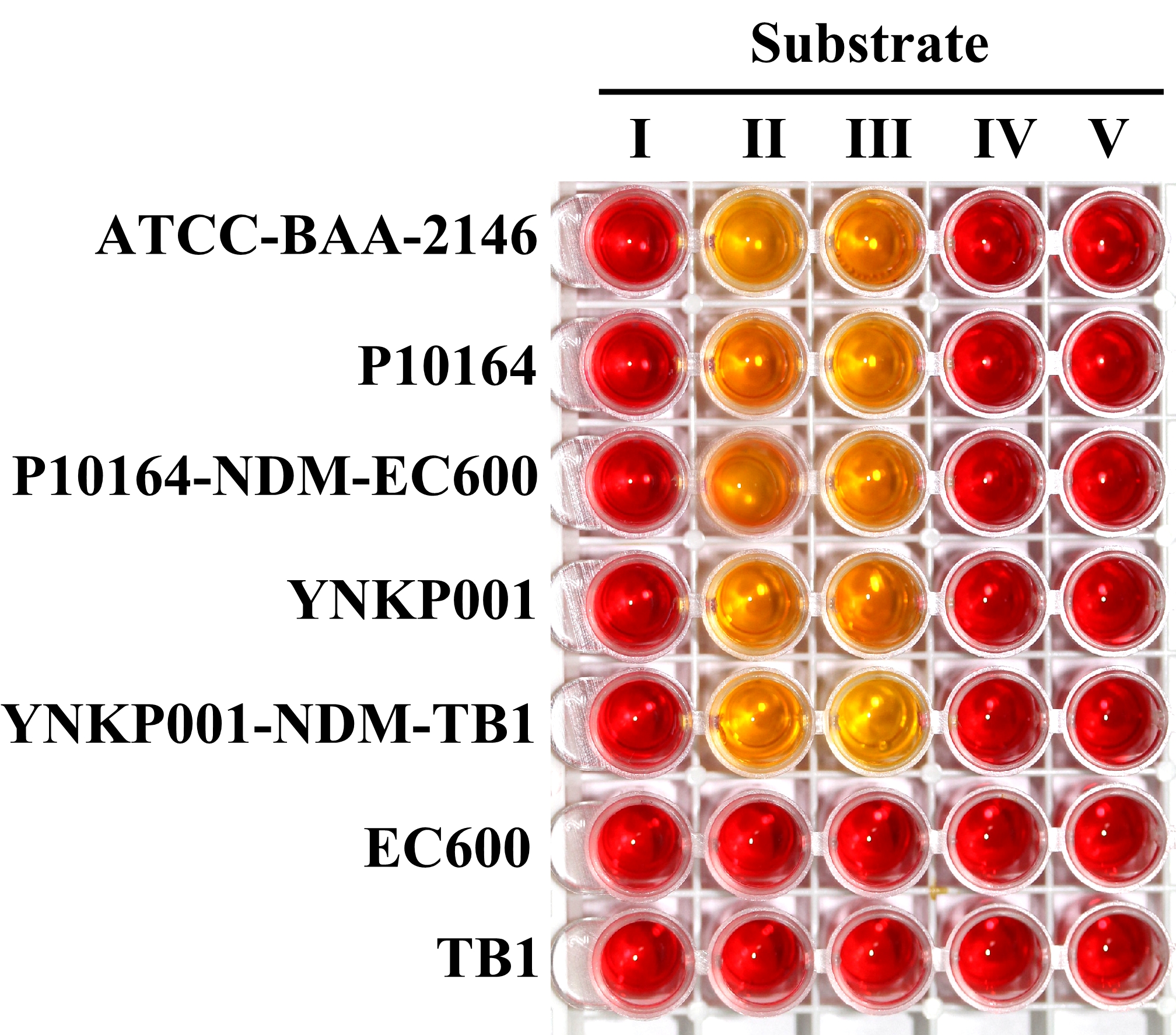

Supplement: Figure S1 — Detection of carbapenemase activity. In the presence of any carbapenemase, relevant carbapenems are hydrolyzed and transformed into its carboxylic form, leading to a pH decrease, which is detected by a color change of phenol red solution (red to yellow-orange). Ambler class A carbapenemases are at least partially inhibited by tazobactam, whereas class B carbapenemases (metallo-ß-lactamases) are inhibited by divalent cation chelators, such as EDTA. There is no available chemical inhibitor for class D carbapenemases. In this study, the blaNDM−1-positive strains R. ornithinolytica YNKP001 and L. adecarboxylata P10164, E. coli YNKP001-NDM-TB1 and P10164-NDM-EC600, and K. pneumoniae ATCC BAA-2146 (positive control) (Rasheed et al., 2013) exhibited class B carbapenemase activity. As expected, E. coli EC600 and DH10B had no carbapenemase activity. [file Image1.JPEG]
