# Supplementary material for: Production of plasmid-encoding NDM-1 in clinical Raoultella ornithinolytica and Leclercia adecarboxylata from China
Source: Front Microbiol. 2015 May 21;6:458. doi: 10.3389/fmicb.2015.00458 (PMC4439573; doi:10.3389/fmicb.2015.00458)
Supplement: Supplementary file 2 [file Table1.DOCX]

**Table S1 PCR detection of carbapenemase and extended-spectrum ß-lactamase (ESBL) genes**

| **ß-lactamase** | | | | **PCR detection** | | | |
| --- | --- | --- | --- | --- | --- | --- | --- |
| **Ambler**  **class** | **Type** | **Subtype** | **Gene** | **Primers** | **AL** | **AT** | **Reference** |
| **Carbapenemases** | | | | | | | |
| A | GES | GES-2, GES-4 to GES-6, GES-11, GES-14, GES-18 | *bla*_GES_ | GES-F: GCTTCATTCACGCACTATT  GES-R: CGATGCTAGAAACCGCTC | 323 | 52 | [[1](#_ENREF_1)] |
|  | KPC | KPC-1 to KPC-15 | *bla*_KPC_ | KPC-F: GTATCGCCGTCTAGTTCTGC  KPC-R: GGTCGTGTTTCCCTTTAGCC | 638 | 56 | [[1](#_ENREF_1)] |
|  | SME | SME-1 to SME-3 | *bla*_SME_ | SME-F1: GAGGAAGACTTTGATGGGAGGAT  SME-R1: TCCCCTCAGGACCGCCAAG | 334 | 52 | [[1](#_ENREF_1)] |
|  | IMI  (NMC-A) | IMI-1 to IMI-3 | *bla*_IMI_ | IMI-F: TGCGGTCGATTGGAGATAAA  IMI-R: CGATTCTTGAAGCTTCTGCG | 399 | 52 | [[1](#_ENREF_1)] |
|  | BIC | BIC-1 | *bla*_BIC_ | BIC-F: TATGCAGCTCCTTTAAGGGC  BIC-R: TCATTGGCGGTGCCGTACAC | 537 | 52 | [[2](#_ENREF_2)] |
| B1 | IMP | IMP-1 to IMP-44 | *bla*_IMP_ | IMP-F: GGAATAGAGTGGCTTAAYTCTC  IMP-R: GGTTTAAYAAAACAACCACC | 232 | 56 | [[2](#_ENREF_2)] |
|  | VIM | VIM-1 to VIM-37 | *bla*_VIM_ | VIM-F: GATGGTGTTTGGTCGCATA  VIM-R: CGAATGCGCAGCACCAG | 390 | 52 | [[2](#_ENREF_2)] |
|  | NDM | NDM-1 to NDM-8 | *bla*_NDM_ | NDM-F: GGTTTGGCGATCTGGTTTTC  NDM-R: CGGAATGGCTCATCACGATC | 621 | 56 | [[2](#_ENREF_2)] |
|  | TMB | TMB-1 to TMB-2 | *bla*_TMB_ | TMB-F: CAAGGAGCTCATTCAAAGG  TMB-R: TTCTAGCGGATTGTGGCCAC |  | 52 | [[3](#_ENREF_3)] |
|  | FIM | FIM-1 | *bla*_FIM_ | FIM-F: GAAGCACATGGAAAACTGGG  FIM-R: GATGGGCGAATGAGACAGC |  | 52 | [[4](#_ENREF_4)] |
|  | SPM | SPM-1 | *bla*_SPM_ | SPM-F: AAAATCTGGGTACGCAAACG  SPM-R: ACATTATCCGCTGGAACAGG | 271 | 52 | [[2](#_ENREF_2)] |
|  | DIM | DIM-1 | *bla*_DIM_ | DIM-F: GCTTGTCTTCGCTTGCTAACG  DIM-R: CGTTCGGCTGGATTGATTTG | 699 | 52 | [[2](#_ENREF_2)] |
|  | GIM | GIM-1 | *bla*_GIM_ | GIM-F: TCGACACACCTTGGTCTGAA  GIM-R: AACTTCCAACTTTGCCATGC | 477 | 52 | [[2](#_ENREF_2)] |
|  | SIM | SIM-1 | *bla*_SIM_ | SIM-F: TACAAGGGATTCGGCATCG  SIM-R: TAATGGCCTGTTCCCATGTG | 570 | 52 | [[2](#_ENREF_2)] |
| B3 | AIM | AIM-1 | *bla*_AIM_ | AIM-F: CTGAAGGTGTACGGAAACAC  AIM-R: GTTCGGCCACCTCGAATTG | 322 | 52 | [[2](#_ENREF_2)] |
|  | SMB | SMB-1 | *bla*_SMB_ | SMB-F: CAGCAGCCATTCACCATCTA  SMB-R: GAAGACCACGTCCTTGCACT | 492 | 52 | [[5](#_ENREF_5)] |
| D | OXA | OXA-23-like | *bla*_OXA-23-like_ | OXA-23-F: GATCGGATTGGAGAACCAGA  OXA-23-R: ATTTCTGACCGCATTTCCAT | 501 | 56 | [[6](#_ENREF_6)] |
|  |  | OXA-24-like | *bla*_OXA-24-like_ | OXA-24-F: GGTTAGTTGGCCCCCTTAAA  OXA-24-R: AGTTGAGCGAAAAGGGGATT | 246 | 52 | [[6](#_ENREF_6)] |
|  |  | OXA-48-like | *bla*_OXA-48-like_ | OXA-48-F: TTGGTGGCATCGATTATCGG  OXA-48-R: GAGCACTTCTTTTGTGATGGC | 744 | 52 | [[7](#_ENREF_7)] |
|  |  | OXA-58-like | *bla*_OXA-58-like_ | OXA-58-F: AAGTATTGGGGCTTGTGCTG  OXA-58-R: CCCCTCTGCGCTCTACATAC | 599 | 56 | [[6](#_ENREF_6)] |
|  |  | OXA-143-like | *bla*_OXA-143-like_ | OXA-143-F: TGGCACTTTCAGCAGTTCCT  OXA-143-R: TAATCTTGAGGGGGCCAACC | 149 | 52 | [[8](#_ENREF_8)] |
|  |  | OXA-235-like | *bla*_OXA-235-like_ | OXA-235-F: TTGTTGCCTTTACTTAGTTGC  OXA-235-R: CAAAATTTTAAGACGGATCG | 768 | 52 | [[9](#_ENREF_9)] |
|  |  | OXA-114 | *bla*_OXA-114_ | OXA-114-F: CGCATCCTGTTCCAGCA  OXA-114-R: GTGCCGGTCTTGCCATAC | 509 | 52 | [[10](#_ENREF_10)] |
| **ESBLs** | | | | | | | |
| A | CTX-M | CTX-M universal | *bla*_CTX-M universal_ | CTX-M-UF: ATGTGCAGYACCAGTAARGT  CTX-M-UR: TGGGTRAARTARGTSACCAGA | 593 | 52 | [[11](#_ENREF_11)] |
|  |  | CTX-M-1 group | *bla*_CTX-M-1 group_ | CTX-M-1GF: AAAAATCACTGCGCCAGTTC  CTX-M-1GR: AGCTTATTCATCGCCACGTT | 415 | 52 | [[12](#_ENREF_12)] |
|  |  | CTX-M-2 group | *bla*_CTX-M-2 group_ | CTX-M-2GF: CGACGCTACCCCTGCTATT  CTX-M-2GR: CCAGCGTCAGATTTTTCAGG | 552 | 52 | [[12](#_ENREF_12)] |
|  |  | CTX-M-8 group | *bla*_CTX-M-8 group_ | CTX-M-8GF: TCGCGTTAAGCGGATGATGC  CTX-M-8GR: AACCCACGATGTGGGTAGC | 666 | 52 | [[12](#_ENREF_12)] |
|  |  | CTX-M-9 group | *bla*_CTX-M-9 group_ | CTX-M-9GF: ATGGTGACAAAGAGAGTGCA  CTX-M-9GR: CCCTTCGGCGATGATTCTC | 869 | 52 | [[13](#_ENREF_13)] |
|  |  | CTX-M-25 group | *bla*_CTX-M-25 group_ | CTX-M-25GF: GCACGATGACATTCGGG  CTX-M-25GR: AACCCACGATGTGGGTAGC | 327 | 52 | [[12](#_ENREF_12)] |
|  | TEM |  | *bla*_TEM_ | TEM-F: CATTTCCGTGTCGCCCTTATTC  TEM-R: CGTTCATCCATAGTTGCCTGAC | 800 | 52 | [[14](#_ENREF_14)] |
|  | SHV |  | *bla*_SHV_ | SHV-F: AGCCGCTTGAGCAAATTAAAC  SHV-R: ATCCCGCAGATAAATCACCAC | 713 | 52 | [[14](#_ENREF_14)] |
|  | GES |  | *bla*_GES_ | GES-ESBL-F: AGTCGGCTAGACCGGAAAG  GES-ESBL-R: TTTGTCCGTGCTCAGGAT | 399 | 52 | [[14](#_ENREF_14)] |
|  | PER |  | *bla*_PER_ | PER-F: GCTCCGATAATGAAAGCGT  PER-R: TTCGGCTTGACTCGGCTGA | 520 | 52 | [[14](#_ENREF_14)] |
|  | VEB |  | *bla*_VEB_ | VEB-F: CATTTCCCGATGCAAAGCGT VEB-R: CGAAGTTTCTTTGGACTCTG | 648 | 52 | [[14](#_ENREF_14)] |
| D | OXA | OXA-1 group | *bla*_OXA-1 group_ | OXA-1-F: GGCACCAGATTCAACTTTCAAG  OXA-1-R: GACCCCAAGTTTCCTGTAAGTG | 564 | 52 | [[14](#_ENREF_14)] |
|  |  | OXA-2 group | *bla*_OXA-2 group_ | OXA-2-F: GACCAAGATTTGCGATCAGCAATGCG  OXA-2-R: CYTTGACCAAGCGCTGATGTTCYACC | 256 | 52 | [[14](#_ENREF_14)] |
|  |  | OXA-10 group | *bla*_OXA-10 group_ | OXA-10-F: CGCCAGAGAAGTTGGCGAAGTAAG  OXA-10-R: GAAACTCCACTTGATTAACTGCGG | 138 | 52 | [[14](#_ENREF_14)] |

AL: amplicon length (bp); AT: annealing temperature (°C)

**References**

1. Hong SS, Kim K, Huh JY, Jung B, Kang MS, Hong SG: Multiplex PCR for rapid detection of genes encoding class A carbapenemases*.* *Annals of laboratory medicine* 32(5), 359-361 (2012).

2. Poirel L, Walsh TR, Cuvillier V, Nordmann P: Multiplex PCR for detection of acquired carbapenemase genes*.* *Diagn Microbiol Infect Dis* 70(1), 119-123 (2011).

3. El Salabi A, Borra PS, Toleman MA, Samuelsen O, Walsh TR: Genetic and biochemical characterization of a novel metallo-beta-lactamase, TMB-1, from an Achromobacter xylosoxidans strain isolated in Tripoli, Libya*.* *Antimicrob Agents Chemother* 56(5), 2241-2245 (2012).

4. Pollini S, Maradei S, Pecile P *et al.*: FIM-1, a new acquired metallo-beta-lactamase from a Pseudomonas aeruginosa clinical isolate from Italy*.* *Antimicrob Agents Chemother* 57(1), 410-416 (2013).

5. Wachino J, Yoshida H, Yamane K *et al.*: SMB-1, a novel subclass B3 metallo-beta-lactamase, associated with ISCR1 and a class 1 integron, from a carbapenem-resistant Serratia marcescens clinical isolate*.* *Antimicrob Agents Chemother* 55(11), 5143-5149 (2011).

6. Woodford N, Ellington MJ, Coelho JM *et al.*: Multiplex PCR for genes encoding prevalent OXA carbapenemases in Acinetobacter spp*.* *Int J Antimicrob Agents* 27(4), 351-353 (2006).

7. Poirel L, Potron A, Nordmann P: OXA-48-like carbapenemases: the phantom menace*.* *J Antimicrob Chemother* 67(7), 1597-1606 (2012).

8. Higgins PG, Lehmann M, Seifert H: Inclusion of OXA-143 primers in a multiplex polymerase chain reaction (PCR) for genes encoding prevalent OXA carbapenemases in Acinetobacter spp*.* *Int J Antimicrob Agents* 35(3), 305 (2010).

9. Higgins PG, Perez-Llarena FJ, Zander E, Fernandez A, Bou G, Seifert H: OXA-235, a novel Class D Beta-Lactamase Involved in Resistance to Carbapenems in Acinetobacter baumannii*.* *Antimicrob Agents Chemother*, (2013).

10. Turton JF, Mustafa N, Shah J, Hampton CV, Pike R, Kenna DT: Identification of Achromobacter xylosoxidans by detection of the bla(OXA-114-like) gene intrinsic in this species*.* *Diagn Microbiol Infect Dis* 70(3), 408-411 (2011).

11. Pagani L, Dell'amico E, Migliavacca R *et al.*: Multiple CTX-M-type extended-spectrum beta-lactamases in nosocomial isolates of Enterobacteriaceae from a hospital in northern Italy*.* *J Clin Microbiol* 41(9), 4264-4269 (2003).

12. Woodford N, Fagan EJ, Ellington MJ: Multiplex PCR for rapid detection of genes encoding CTX-M extended-spectrum (beta)-lactamases*.* *J Antimicrob Chemother* 57(1), 154-155 (2006).

13. Barguigua A, El Otmani F, Talmi M *et al.*: Characterization of extended-spectrum beta-lactamase-producing Escherichia coli and Klebsiella pneumoniae isolates from the community in Morocco*.* *J Med Microbiol* 60(Pt 9), 1344-1352 (2011).

14. Dallenne C, Da Costa A, Decre D, Favier C, Arlet G: Development of a set of multiplex PCR assays for the detection of genes encoding important beta-lactamases in Enterobacteriaceae*.* *J Antimicrob Chemother* 65(3), 490-495 (2010).
